# Remote ischemic perconditioning attenuates ischemia/reperfusion‐induced downregulation of AQP2 in rat kidney

**DOI:** 10.14814/phy2.12865

**Published:** 2016-07-12

**Authors:** Marie Louise V. Kristensen, Casper Kierulf‐Lassen, Per Mose Nielsen, Søren Krag, Henrik Birn, Lene N. Nejsum, Rikke Nørregaard

**Affiliations:** ^1^ Department of Clinical MedicineAarhus UniversityAarhusDenmark; ^2^ Department of PathologyAarhus UniversityAarhusDenmark; ^3^ Department of BiomedicineAarhus UniversityAarhusDenmark; ^4^ Department of Renal MedicineAarhus UniversityAarhusDenmark

**Keywords:** AQP2, I/R injury, Na–K‐ATPase, remote ischemic perconditioning

## Abstract

Renal ischemia/reperfusion (I/R) can lead to impaired urine concentration ability and increased fractional excretion of sodium (FeNa). Local ischemic *pre*conditioning improves renal water and sodium handling after I/R injury. Here, we investigate whether remote ischemic *per*conditioning (rIPeC) prevents dysregulation of renal water and salt handling in response to I/R injury and mechanisms that may be involved. Rats were subjected to right nephrectomy and randomized into a sham group or an I/R group. In the I/R group, rats were subjected to 37 min of renal ischemia and 3 days of reperfusion. rIPeC was applied to the abdominal aorta. Blood and urine were collected on day 3 postoperatively for clearance studies. The expression of aquaporins (AQPs) and the sodium transporter Na–K‐ATPase were analyzed using immunoblotting and immunohistochemistry. I/R injury resulted in polyuria, increased FeNa, and decreased urine osmolality compared to sham rats. rIPeC attenuated the increase in FeNa and the decrease in urine osmolality. Expression of AQP1, AQP2, phosphorylated AQP2 (pAQP2), and Na–K‐ATPase was downregulated in I/R rats. rIPeC attenuated the reductions in AQP2 and pAQP2 expression. Immunohistochemistry revealed decreased labeling of Na–K‐ATPase in the outer medulla in I/R kidneys compared to kidneys from sham and I/R + rIPeC rats. After renal ischemia, the expression of Na–K‐ATPase was substantially reduced in the outer medullary thick ascending limb. In conclusion, our data suggest that rIPeC might prevent dysregulation of renal water and salt handling via regulation of AQP2 expression and phosphorylation as well as via regulation of Na–K‐ATPase expression in I/R rat kidneys.

## Introduction

Renal ischemia/reperfusion (I/R) injury, which can cause acute kidney injury (AKI), is a common clinical problem associated with prolonged hospitalization, high mortality, and risk of progression to chronic kidney disease. It may be associated with renal transplantation, cardiovascular surgery, and hypotension with subsequent restoration of renal perfusion (Ali et al. 2007; Bonventre and Yang 2011; Case et al. 2013; Coca et al. 2012). Severe reduction in glomerular filtration rate (GFR), decreased urinary concentration ability, and impairment of tubular reabsorption of water and sodium are all the characteristics of I/R (Gong et al. 2004; Kwon et al. 1999). Impairment of tubular reabsorption of water and sodium may occur because of damage to the renal tubular epithelia, leading to functional and structural changes. The proximal tubule (PT, especially the S3 segment) and the outer medullary thick ascending limb (TAL) are particularly susceptible to I/R (Gong et al. 2004; Venkatachalam et al. 1978). In addition, several studies have demonstrated defects in collecting duct (CD) water and sodium handling in the postischemic kidney (Fernández‐Llama et al. 1999; Gong et al. 2004; Kwon et al. 1999). Dysregulation of aquaporins (AQPs) and sodium transporter proteins in the renal tubules is believed to be involved in the alterations in water and sodium handling following I/R injury (Fernández‐Llama et al. 1999; Gong et al. 2004; Kwon et al. 1999).

AQPs are transmembrane water channels that facilitate the movement of water across cell membranes. AQP1 is constitutively expressed in the PT and plays an important role in water reabsorption by this kidney segment (Nielsen et al. 2002). In the CD, AQP2 is involved in water reabsorption at the apical membranes (Fushimi et al. 1993). The transcription and phosphorylation of AQP2, and thereby its trafficking to the apical membranes, are regulated by vasopressin. AQP2 has several phosphorylation sites, and phosphorylation at serine 256 (Ser256) is crucial for trafficking (Fushimi et al. 1997; Lu et al. 2008). The expression of these AQPs has been shown to be markedly reduced in rats exposed to I/R injury (Gong et al. 2004; Kwon et al. 1999). Tubular water reabsorption is driven by the active reabsorption of sodium via several sodium transport proteins, such as Na–K‐ATPase (Kokko and Rector 1972). Na–K‐ATPase localizes to the basolateral membranes in polarized epithelial cells, mainly in the PT and TAL (Jorgensen 1980) and studies have likewise shown decreased Na–K‐ATPase expression in response to I/R injury (Gong et al. 2004).

It has been demonstrated that ischemic conditioning (IC) improves creatinine clearance (CrCl) and attenuates polyuria and fractional excretion of sodium (FeNa) in I/R injury (Ogawa et al. 2000; Yamashita et al. 2003). The protective effect of IC was first described by Murry et al., who showed that short periods of nonharming occlusions of myocardial blood flow prior to sustained ischemia protected against I/R injury in the heart and reduced myocardial infarct size (Murry et al. 1986). Subsequently, the effect of IC has been intensely investigated in various organs, including the kidney. Protection against renal I/R injury may also be induced by remote IC, which involves applying brief periods of alternating ischemia and reperfusion to an organ or tissue remote from the kidney (Gassanov et al. 2014; Gho et al. 1996). Several mechanisms have been suggested to be implicated in the effects of IC (Gassanov et al. 2014), but so far the molecular mechanisms underlying the effects of IC on renal water and sodium handling remain unknown. This study was designed to investigate whether remote ischemic *per*conditioning (rIPeC) applied during sustained ischemia to the kidney prevents renal dysfunction and improves water and sodium handling following I/R. To further elucidate the mechanisms involved, we investigated the effect of rIPeC on the expression and phosphorylation of AQPs and expression of the Na–K‐ATPase in postischemic kidneys. Our hypothesis is that remote ischemic *per*conditioning (rIPeC) improves renal water and sodium handling by stimulating the expression of AQPs and Na–K‐ATPase in postischemic kidneys.

## Materials and Methods

### Experimental animals

Studies were performed on male Munich‐Wistar rats initially weighing 220–250 g. The experimental protocol was approved by the Animal Experiments Inspectorate, under the Danish Veterinary and Food Administration (Approval no. 2013‐15‐2934‐00810), and all procedures were carried out in accordance with the Danish National Guidelines for care and handling of experimental animals. Rats were kept in cages in a 12:12 h light–dark cycle, a temperature of 21± 2°C, and a humidity of 55 ± 5%. Animals had ad libitum access to a standard rodent diet (Altromin, Lage, Germany) and tap water.

### Surgical procedures

Rats were anesthetized with sevoflurane and placed on a heating pad to maintain a rectal temperature of 36–37°C during surgery. 7 days prior to I/R injury, the right kidney was removed through an abdominal midline incision. The rats were then reopened and the left renal artery and the abdominal aorta were carefully dissected. A nontraumatic clamp was placed around the renal artery and closed for 37 min. Occlusion of blood supply was confirmed by color change in the kidney. After 37 min, the clamp was removed and reperfusion was visually confirmed. To prevent arterial spasms impeding reperfusion of the kidney, a drop of lidocaine was applied to the area around the renal artery a few minutes prior to removal of the clamp. The incision was then closed. The total time of anesthesia was kept similar in all groups to avoid the possible confounding effects of different anesthesia times. rIPeC was performed immediately after clamping of the renal artery by clamping of the abdominal aorta with a nontraumatic clamp. The aorta was occluded for 5 min, blocking perfusion of the hind limbs and pelvic organs, after which the clamp was removed to allow reperfusion for 5 min. This cycle was repeated four times (4 × 5 min rIPeC). Age‐ and time‐matched sham‐operated controls were prepared in parallel with each I/R and I/R + rIPeC group as follows in the protocol.

#### Protocol

**Figure 1:**
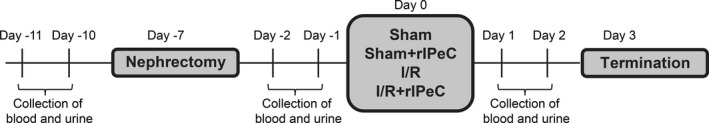
Diagram of study design. Rats were subjected to nephrectomy 7 days before ischemia and reperfusion (I/R) injury. I/R injury was established by temporary renal artery occlusion for 37 min followed by reperfusion. Rats were monitored over the following 3 days. Remote ischemic perconditioning (rIPeC) was performed after clamping of the renal artery by clamping of the abdominal aorta. The aorta was occluded for 5 min, after which the clamp was removed to allow reperfusion for 5 min. This cycle was repeated four times (4 × 5 min rIPeC). Sham‐operated rats were operated without occlusion.

The effect of I/R injury and IC was evaluated using a unilateral nephrectomy model allowing evaluation of the function of the injured kidney through blood sampling and total urine collection. Nephrectomy of the right kidney was performed 1 week prior to surgical interventions on the left kidney (Fig. 1). Rats were randomized into four groups: (1) sham‐operated animals (sham group, *n *=* *12); (2) sham‐operated animals subjected to rIPeC (sham + rIPeC group, *n *=* *4); (3) 37‐min renal ischemia followed by 3 days reperfusion (I/R group, *n *=* *11); and (4) 37‐min renal ischemia followed by 3 days reperfusion combined with rIPeC (I/R + rIPeC group, *n *=* *12). For urine sampling, rats were placed in metabolic cages for 24 h and blood and urine were collectively sampled on study day 10 (before unilateral nephrectomy), day 1 (before I/R injury), and days 2 and 3 (after I/R injury) (Fig. 1). Rats were killed for either immunoblotting (*n *=* *4–8 in each group) or immunohistochemistry (*n *=* *4 in each group).

### Renal functional parameters

Plasma creatinine, urea, Na^+^ and K^+^, and urinary creatinine and urea were measured using a Roche cobas 6000 analyzer (Roche Diagnostics, Hvidovre, Denmark). Urinary Na^+^ and K^+^ were measured by standard flame photometry (FCM6341; Eppendorf, AH Diagnostics, Aarhus, Denmark). Plasma and urine osmolality were measured with vapor pressure osmometers (Osmomat 030‐D; Gonotec; and Multi‐Sample Osmometer, Advanced Instruments, Praestoe, Denmark, respectively). CrCl and FeNa were calculated on the basis of these measurements.

### Gel electrophoresis and western blotting

The kidney was rapidly removed and dissected into cortex + outer medulla (OM) and inner medulla (IM). The kidney zones were homogenized in RIPA buffer supplemented with Protease Inhibitor Cocktails 2 and 3 and Complete Mini‐Protease Inhibitor Tablet (Roche Diagnostics). The tissue was homogenized for 30 sec at 60 Hz in a TissueLyser LT (Qiagen, Copenhagen, Denmark) followed by centrifugation at 1000*g* for 10 min at 4°C. The protein concentration was measured in the supernatant using a Pierce BCA Protein Assay Kit (Roche Diagnostics). Proteins were separated by SDS‐PAGE on an 8–16% polyacrylamide gel (Criterion TGX Stain‐Free Gels and Criterion TGX Precast Midi Protein Gels, Bio‐Rad, Copenhagen, Denmark). After gel activation on a ChemiDoc MP imager (Bio‐Rad), the proteins were transferred to a nitrocellulose membrane using a Trans‐Blot^®^ Turbo^™^ Transfer System (Bio‐Rad). Total protein was measured on the ChemiDoc MP imager and the membranes were blocked in 5% skim milk in PBS‐Tween (PBS‐T). Membranes were incubated with primary antibody overnight at 4°C, washed in PBS‐T, and incubated with secondary antibody for one hour at room temperature. The blots were visualized on the ChemiDoc MP imager using the detection reagent Amersham ECL‐Prime (GE Healthcare, Brøndby, Denmark) or with ECL (GE Healthcare) using film. Intensity of the total protein amount and of protein bands of interest were quantified using Image Lab software (Bio‐Rad). Protein bands of interest were normalized to the total protein measurements as described in Gürtler et al. (2013) and Posch et al. (2013).

### Histology and immunohistochemistry

Kidneys were fixed by retrograde perfusion via the abdominal aorta with 4% paraformaldehyde in 0.01 mol/L PBS. Next, kidneys were fixed for 2 h and washed in PBS. The fixed kidneys were then dehydrated, embedded in paraffin, and cut into 2‐*μ*m sections on a rotary microtome (Leica Microsystems A/S, Herlev, Denmark).

To evaluate morphological damage, paraffin‐embedded sections containing all kidney zones were stained with periodic acid–Schiff (PAS) stain according to the manufacturer's protocol (Sigma‐Aldrich, Brøndby, Denmark). Blinded evaluation by conventional light microscopy was carried out by a skilled pathologist at the Department of Pathology, Aarhus University Hospital.

For immunoperoxidase labeling, endogenous peroxidase was blocked in 35% H_2_O_2_ dissolved in methanol. Afterward, the sections were boiled in TEG buffer (1 mmol/L Tris, 0.5 mmol/L ethylene glycol tetraacetic acid, pH 9.0) for 10 min for antigen retrieval. Nonspecific binding of immunoglobulin was prevented by incubating the sections in 50 mmol/L NH_4_Cl for 30 min followed by blocking in PBS supplemented with 1% bovine serum albumin (BSA), 0.2% gelatin, and 0.05% saponin. Sections were incubated with primary antibodies diluted in 0.1% BSA and 0.3% Triton X‐100 at 4°C overnight in a humidity chamber. After being rinsed in PBS supplemented with 0.1% BSA, 0.2% gelatin, and 0.05% saponin for 3 × 10 min, the sections for immunoperoxidase labeling were incubated for one hour at room temperature with horseradish peroxidase‐conjugated secondary antibody. After rinsing, the sections were incubated in 3,3′‐diaminobenzidine for 10 min in order to visualize the peroxidase, and counterstained in Mayer's hematoxylin. Conventional light microscopy was performed using an Olympus BX50 microscope. For immunofluorescence staining, double labeling was performed using primary antibodies against AQP2, Na–K‐ATPase, and Tamm–Horsfall protein (THP). The labeling was visualized with Alexa 568‐, Alexa 488‐, and Alexa 594‐conjugated secondary antibodies, respectively. Imaging was performed using a fluorescence microscope (Olympus BX61) and xcellence Rt software (Olympus Soft Imaging Solution GMBH, Münster, Germany). For quantification of fluorescence staining intensity, images were imported into ImageJ (Schneider et al. 2012). Tubules were traced manually and fluorescence intensity was measured. After background subtraction, intensity in relation to tubule area (arbitrary units) was calculated as the average Na–K‐ATPase intensity per tubule area (*n *=* *40–50 tubules pr. group). THP staining was used to determine TAL (Fig. 6) and AQP2 staining was used to determine collecting ducts (Fig. 7). Blinded evaluation of all the tubules was performed in order to score them as injured or noninjured. Surface plots were generated in ImageJ.

### Primary antibodies

For protein identification in western blotting (WB), immunohistochemistry, and immunofluorescence experiments, the following primary antibodies were used: anti‐AQP1 (Terris et al. 1996), anti‐AQP2 (DiGiovanni et al. 1994), anti‐pAQP2 (Christensen et al. 2000) were used for WB and immunohistochemistry; anti‐Na–K‐ATPase (*α*1‐subunit) (Kashgarian et al. 1985) was used for WB; anti‐Na–K‐ATPase (*α*1‐subunit) (Merck Millipore, Hellerup, Denmark) was used for immunohistochemistry and immunofluorescence; anti‐Hsp70 and anti‐THP (Cayman Chemical, AH Diagnostics, Denmark) were used for immunofluorescence.

### Na–K‐ATPase activity measurements

For the ATPase activity assay, the microsomal fraction of the renal cortex + OM was isolated by the method of Sarkar (2002) and Jorgensen (1974, 1980) with slight modification. Briefly, tissue was homogenized (approx. 10% w/v) using a glass homogenizer in an ice‐cold 0.32 mol/L sucrose and centrifuged at 1000*g* for 10 min to remove cell debris and nuclei. The supernatant was layered over 1.2 mol/L sucrose and centrifuged at 34,000*g* for 50 min at 4°C. The upper layer of the pellet was resuspended in an ice‐cold bidistilled water and layered on 0.8 mol/L sucrose and centrifuged at 34,000*g* for 30 min at 4°C. The pellet was resuspended in an ice‐cold 5 mmol/L imidazole–HCl buffer, pH 7.4 and used immediately for assay.

Na–K‐ATPase activity was measured using a high‐sensitivity colorimetric ATPase assay kit following the manufacturer's instruction (Innova Biosciences, AH Diagnostics, Aarhus, Denmark). The microsomes suspension was incubated with the reaction buffer containing (final concentrations) 50 mmol/L Tris, 2.5 mmol/L MgCl_2_, and 0.5 mmol/L ATP, pH 7.4 in the absence and in the presence of 1 mmol/L ouabain for 10 min at 37°C. The amount of inorganic phosphate (P_i_) released was quantified colorimetrically at 620 nm and the protein content was measured with Pierce BCA Protein Assay Kit. The specific activity of Na–K‐ATPase was calculated by subtracting the ouabain‐insensitive activity from the overall activity (in the absence of ouabain) and expressed as *μ*mol P_i_ liberated from ATP by 1 *μ*g of protein per min.

### Statistical analysis

A normal distribution within each group for each parameter was verified by QQ‐plots. The homogeneity of variances was tested using Bartlett's test and, if equal, groups were compared by two‐way ANOVA followed by an uncorrected Fisher's LSD post hoc test. If variances were not equal, groups were compared by a nonparametric Kruskal–Wallis test and Dunn's correction. Changes with time were compared by first calculating delta values within groups, which were then compared using the tests as described above. *P *<* *0.05 was considered significant. Data are presented as mean ± SEM.

## Results

### rIPeC attenuates dysregulation of renal water and sodium handling after I/R injury

As shown in Table 1, the I/R group showed a 6% decrease in body weight compared with the sham group. Kidneys subjected to I/R were visibly swollen and weighed significantly more than sham kidneys. There was no significant effect of rIPeC on body or kidney weight in rats subjected to I/R. Plasma creatinine levels and blood urea nitrogen (BUN) were significantly increased in the I/R group when compared with the sham‐operated group. Consistent with this, CrCl was decreased after I/R injury, indicating that renal I/R resulted in acute renal insufficiency. rIPeC treatment was associated with a nonsignificant lowering of plasma creatinine and BUN and a greater CrCl when compared to nontreated I/R rats. FeNa was increased in the I/R group compared to the sham group, and this increase was significantly attenuated by rIPeC treatment (Table 1).

**Table 1:** Changes in renal function 3 days after release of 37‐min unilateral renal ischemia with or without remote ischemic perconditioning (rIPeC)

	Sham	Sham+rIPeC	I/R	I/R+rIPeC
Body weight (g)	249.6 (±3.2)	257.0 (±3.2)	235.6 (±5.5)^a^	244.8 (±3.2)
Kidney weight (pct. of body weight)	4.7 (±0.1)	4.6 (±0.1)	8.6 (±0.3)^a^	8.0 (±0.4)^b^
Water intake (mL)	31.8 (±0.7)	29.5 (±2.1)	38.2 (±4.2)	36.7 (±3.3)
Food intake (g)	16.1 (±1.3)	16.6 (±0.3)	8.6 (±1.3)^a^	9.6 (±1.1)^b^
Plasma creatinine (*μ*mol/L)	27.3 (±1.3)	24.8 (±1.4)	118.6 (±19.7)^a^	88.4 (±14.5)^b^
BUN (mmol/L)	9.5 (±1.2)	10.1 (±1.8)	25.0 (±4.1)^a^	18.1 (±2.4)
Creatinine clearance (mL/min/kg)	5.2 (±0.6)	6.0 (±0.3)	1.6 (±0.2)^a^	2.1 (±0.3)^b^
FeNa(%)	0.3 (±0.0)	0.2 (±0.0)	0.9 (±0.2)^a^	0.4 (±0.1)^c^

Values are mean ± SEM. BUN, blood urea nitrogen.

a *P *< 0.05 compared to sham group.

b *P* < 0.05 compared to sham+rIPeC group.

c *P* < 0.05 compared to I/R group.

**Figure 2:**
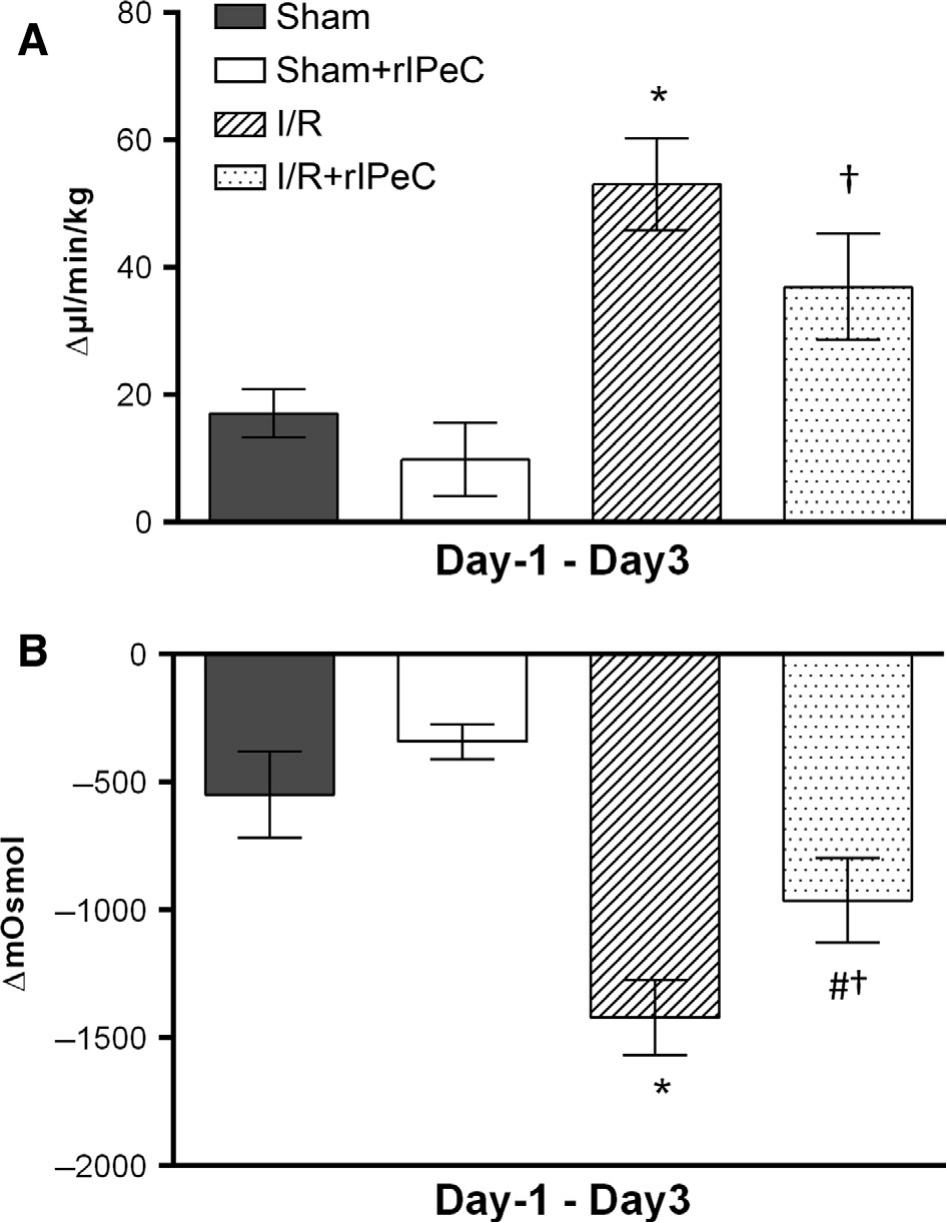
Effects of remote ischemic perconditioning (rIPeC) on changes in urine output and urine osmolality after ischemia and reperfusion (I/R) injury. Changes over time are calculated from day −1 to day 3. (A) Urine output. I/R rats had a significant increase in urine output over the course of the study, and this was unaltered by rIPeC treatment. (B) Urine osmolality. Urine osmolality was significantly decreased in I/R rats and this decrease was attenuated by rIPeC treatment. Data are means ± SEM, *n *=* *4–8 rats per group. *P <* *0.05 compared to the sham group, ^†^ *P *<* *0.05 compared to the sham + rIPeC group, ^#^ *P *<* *0.05 compared to the I/R group.

Urine output increased in the I/R group compared with the sham group when measured over time, consistent with a decrease in urine osmolality (Fig. 2A–B). Administration of rIPeC significantly attenuated the I/R‐induced decrease in urine osmolality with time (Fig. 2B). In addition, urine output was reduced in the I/R rats exposed to rIPeC, but this did not reach statistical significance (*P *=* *0.09) (Fig. 2A). Taken together, these data indicate that rIPeC might play a role in the regulation of altered renal water and sodium handling in response to renal I/R.

### rIPeC attenuates downregulation of AQP2 and pAQP2, but not AQP1, after I/R injury

**Figure 3:**
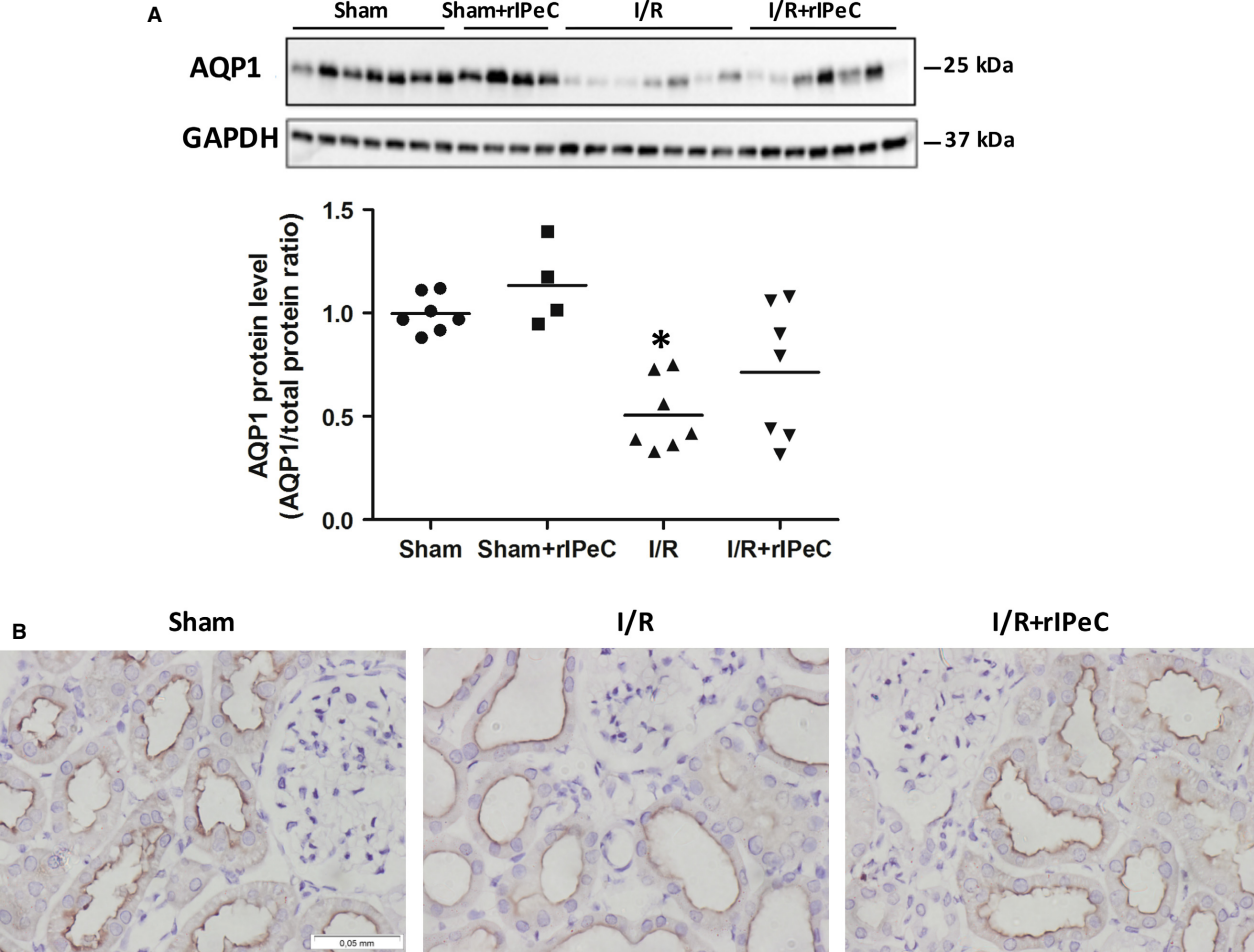
Effects of remote ischemic perconditioning (rIPeC) on aquaporin‐1 (AQP1) expression after ischemia and reperfusion (I/R) injury. (A) Western blot analysis of AQP1 expression in tissue from the cortex and outer medulla (C + OM). (B) AQP1 immunoreactivity in cortex of I/R rats treated with rIPeC. Data are means ± SEM, *n *=* *4–8 rats per group for the western blot analysis and *n *=* *4 rats per group for the immunohistochemical analysis. *P <* *0.05 compared to the sham group. OM, outer medulla.

AQP1 protein expression was downregulated in postischemic kidneys compared to sham‐operated kidneys (Fig. 3A). Immunohistochemistry confirmed that AQP1 labeling was weaker in the apical plasma membranes of cortical PT from I/R kidneys when compared to kidneys from sham‐operated rats (Fig. 3B). rIPeC treatment did not change AQP1 expression or localization in the postischemic kidneys. Additionally, AQP1 levels in ischemic kidneys subjected to rIPeC were also not significantly different from treated controls (Fig. 3A–B).

**Figure 4:**
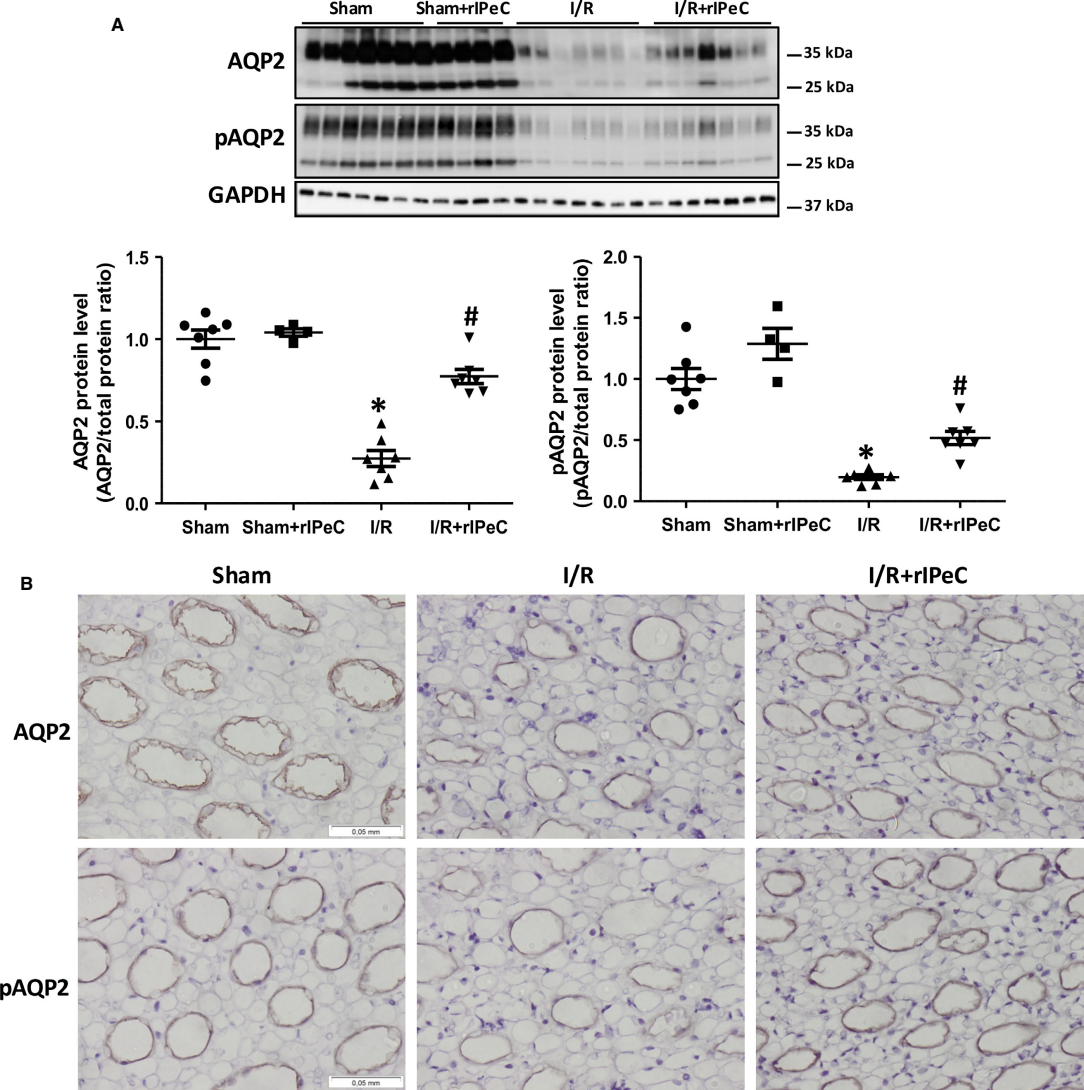
Effects of remote ischemic perconditioning (rIPeC) on aquaporin‐2 (AQP2 and phosphorylated AQP2) expression after ischemia and reperfusion (I/R) injury. (A) Western blot analysis of AQP2 and Ser256‐phosphorylated AQP2 (pAQP2) expression in tissue from the inner medulla (IM) in I/R rats treated with rIPeC. (B) AQP2 and pAQP2 immunoreactivity in the IM of I/R rats treated with rIPeC. Data are means ± SEM, *n *=* *4–8 rats per group for the western blot analysis and *n *=* *4 rats per group for the immunohistochemical analysis. *P <* *0.05 compared to the sham group. ^#^ *P *<* *0.05 compared to the I/R group.

As shown in Fig. 4A, AQP2 and pAQP2 protein expression was markedly reduced in kidneys from I/R rats (Fig. 4A), consistent with reduced labeling of both AQP2 and pAQP2 in the apical membranes of the inner medullary CD, when compared with kidneys from sham‐operated rats (Fig. 4B). Administration of rIPeC attenuated the downregulation of both AQP2 and pAQP2 (Fig. 4A) in line with stronger CD labeling for AQP2 and pAQP2 when compared to I/R only (Fig. 4B).

### rIPeC affects Na–K‐ATPase expression in the OM after I/R injury

**Figure 5:**
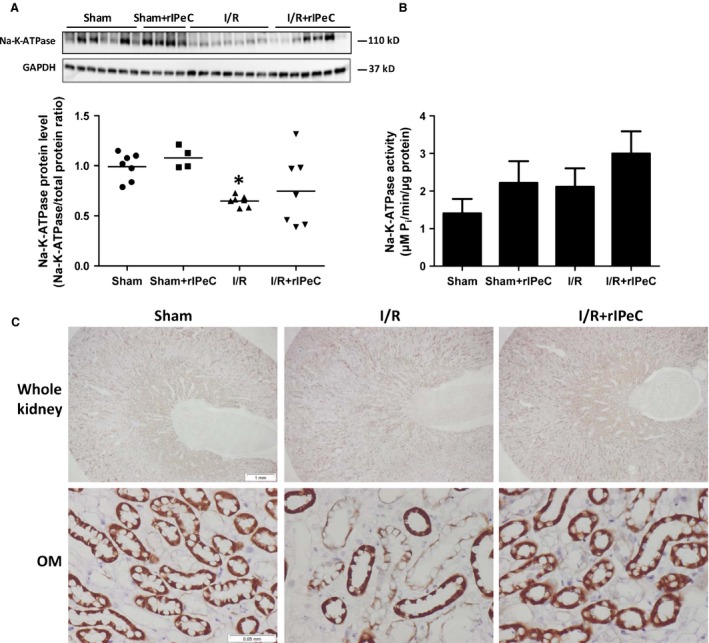
Effects of remote ischemic perconditioning (rIPeC) on Na–K‐ATPase expression and activity after ischemia and reperfusion (I/R) injury. (A) Western blot analysis of Na–K‐ATPase expression in tissue from the cortex and outer medulla (C + OM) in I/R rats treated with rIPeC. (B) Na–K‐ATPase activity measurements in tissue from the cortex and outer medulla (C + OM) in I/R rats treated with rIPeC. (C) Na–K‐ATPase immunoreactivity in whole kidney and outer medulla of I/R rats treated with rIPeC. Data are means ± SEM, *n *=* *4–8 rats per group for the western blot analysis and *n *=* *4 rats per group for the immunohistochemical analysis. *P <* *0.05 compared to the sham group.

Western blotting analysis demonstrated the reduced Na–K‐ATPase expression in the cortex and OM in the I/R rats compared to the sham‐operated rats (Fig. 5A), whereas Na–K‐ATPase activity was not significantly changed between the two groups (Fig. 5B). rIPeC administration tended to increase both Na–K‐ATPase protein levels and activity when compared to I/R, although it did not reach statistical significance (Fig. 5A–B). Immunohistochemical analysis revealed reduced labeling of Na–K‐ATPase in the OM of postischemic kidneys compared to the sham‐operated rats (Fig. 5C). Interestingly, in the rIPeC‐treated I/R kidneys, Na–K‐ATPase staining seemed substantially stronger in the OM when compared to untreated I/R kidneys (Fig. 5C).

**Figure 6:**
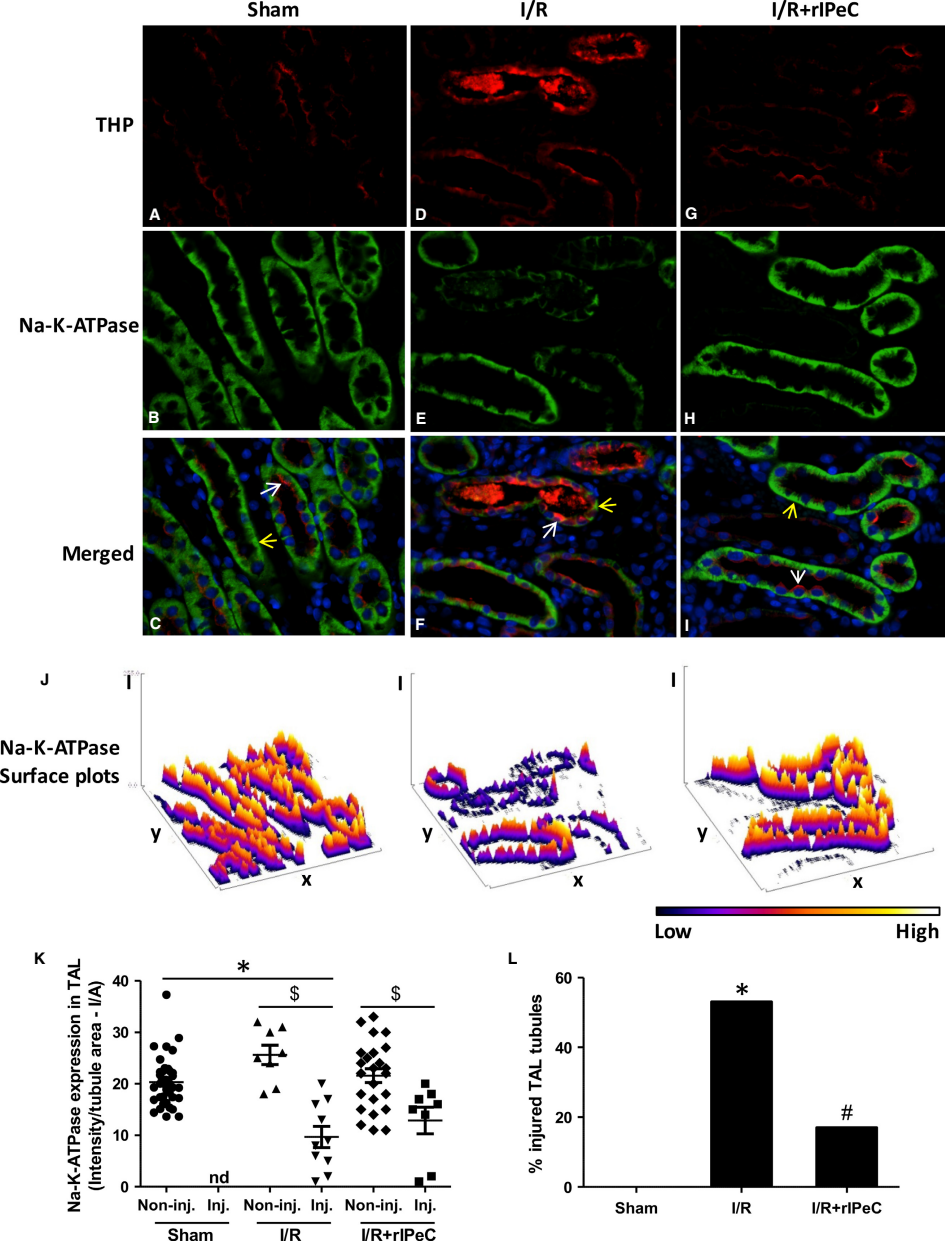
Double immunofluorescence labeling of Na–K‐ATPase and THP in outer medullary TAL in kidneys of sham‐operated, ischemia and reperfusion (I/R), and remote ischemic perconditioning (rIPeC)‐treated I/R rats. Whole kidney sections were incubated with antibodies against Na–K‐ATPase and THP. Labeling was visualized with Alexa 488 (green) and Alexa 594 (red), respectively. In sham‐operated kidneys, abundant basolateral Na–K‐ATPase (green) and apical THP (red) labeling was seen on plasma membrane domains of TAL cells in the outer medulla (OM) (A–C). In I/R rats, labeling of Na–K‐ATPase was reduced and THP staining was strong in TAL with extension into the lumen (D–F), which was not apparent in rIPeC + I/R rats (G–I). White arrows mark the THP staining in TAL, whereas the yellow arrows mark the Na–K‐ATPase staining (C, F, I). (J) Surface blots of Na–K‐ATPase immunofluorescence intensity. (K) Mean values of intensity in TAL tubules of Na–K‐ATPase showed decreased Na–K‐ATPase expression in injured TAL in the ischemic kidney compared to the sham kidneys. rIPeC treatment of I/R rats tend to increase Na–K‐ATPase expression to a level not significantly different from controls. Data are means ± SEM, *n *=* *3–4 rats per group, nd, none detected. (L) Percent of injured TAL tubules relative to the total amount of TAL tubules. *P <* *0.05 compared to the sham group. ^$^ *P *<* *0.05 noninjured TAL tubules versus injured TAL tubules. ^#^ *P *<* *0.05 compared to rIPeC + I/R group. OM, outer medulla; TAL, thick ascending limb; THP, Tamm–Horsfall protein.

To further study in which tubular sections Na–K‐ATPase was regulated in OM in response to I/R and rIPeC, we performed double immunofluorescence labeling of Na–K‐ATPase together with THP, a TAL‐specific marker and AQP2, respectively. Segment‐specific staining and quantification revealed decreased Na–K‐ATPase expression in the outer medullary‐injured TAL in rats exposed to I/R compared to sham‐operated rats (Fig. 6K). Correspondingly, rIPeC‐treated I/R rats showed increased Na–K‐ATPase expression in injured TAL, but this did not reach statistical significance. In addition, Na–K‐ATPase expression in TAL in ischemic kidneys subjected to rIPeC was not different from controls (Fig. 6K). Tubules from kidneys exposed to I/R, showing strong cytoplasmic labeling for THP with frequent extension into the lumen, were only weakly positive for Na–K‐ATPase indicating injured tubules. This was not apparent in sham and I/R + rIPeC kidneys where the THP expression was mainly confined to the apical domains of TAL with lesser extension into the lumen (Fig. 6A–I). The percent of injured TAL tubules was 53% in the I/R rats, whereas only 17% of injured tubules was detected in I/R rats subjected to rIPeC (Fig. 6L).

**Figure 7:**
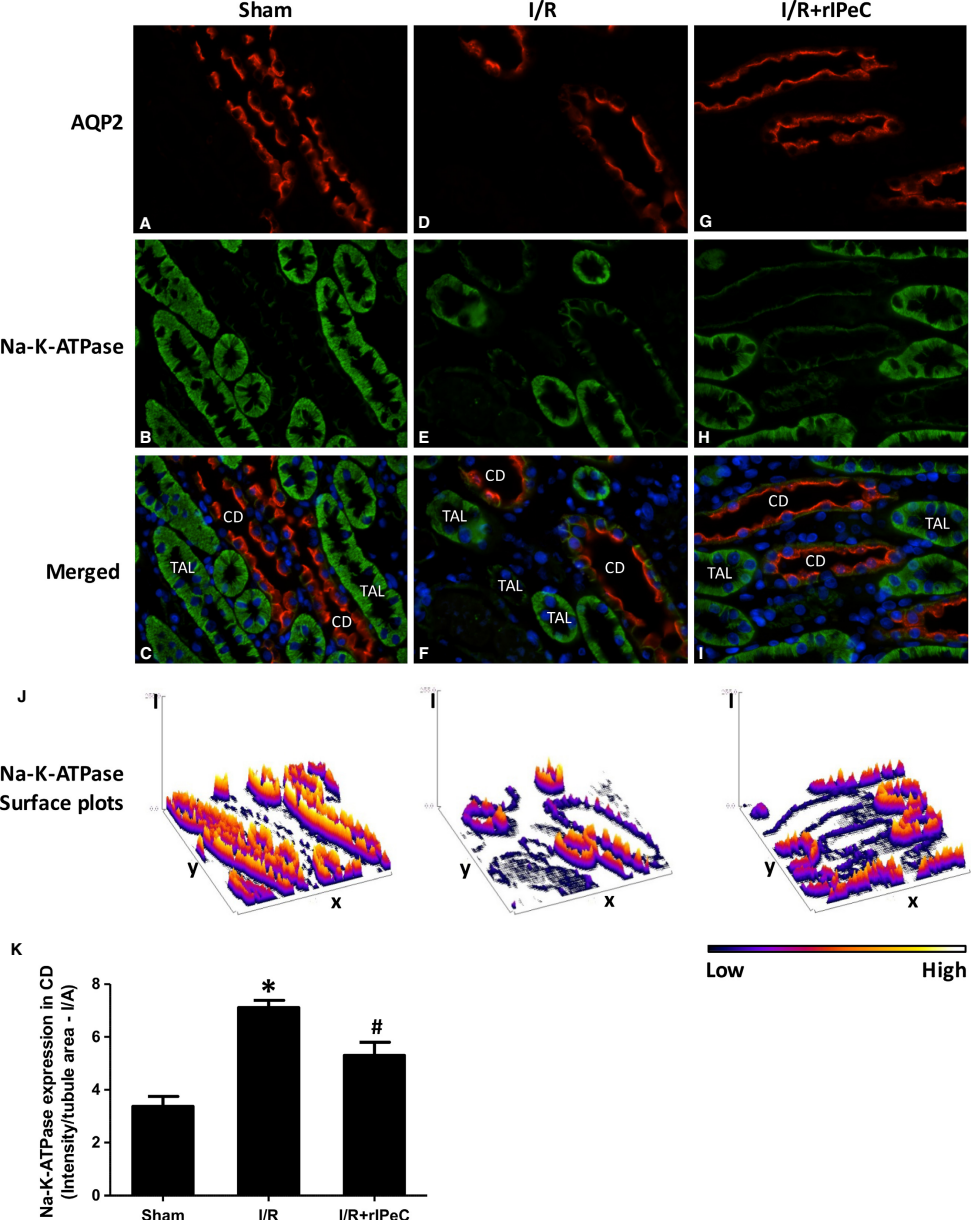
Double immunofluorescence labeling of Na–K‐ATPase and aquaporin‐2 (AQP2) in outer medullary collecting ducts in kidneys of sham‐operated, ischemia and reperfusion (I/R), and remote ischemic perconditioning (rIPeC)‐treated I/R rats. Whole kidney sections were incubated with antibodies against Na–K‐ATPase and AQP2. Labeling was visualized with Alexa 488 (green) and Alexa 564 (red), respectively. In sham‐operated kidneys, basolateral Na–K‐ATPase (green) and apical AQP2 (red) labeling was seen on plasma membrane domains of CD cells in the OM (A–C). In I/R rats, labeling of Na–K‐ATPase was more intense in the AQP2‐positive cells (D–F), which was not apparent in rIPeC + I/R rats (G–I). (J) Surface blots of Na–K‐ATPase immunofluorescence intensity. (K) Mean values of intensity in CD tubules of Na–K‐ATPase showed increased Na–K‐ATPase expression in CD in ischemic kidneys compared to kidneys from sham and rIPeC + I/R rats. Data are means ± SEM, *n *=* *3–4 rats per group. *P <* *0.05 compared to the sham group. ^#^ *P *<* *0.05 compared to rIPeC + I/R group. OM, outer medulla; CD, collecting duct.

Figure 7 shows quantification of Na–K‐ATPase fluorescence staining intensity in AQP2‐positive CD tubules and in TAL in OM. Na–K‐ATPase expression was reduced in the CD compared to TAL in all groups (I/A in tubules in sham rats: CD: 3.4 ± 0.4 vs. TAL: 21.4 ± 0.7). In addition, CD cells from rats exposed to I/R injury had increased Na–K‐ATPase expression which was reduced in sham‐operated rats and I/R rats exposed to rIPeC (Fig. 7K).

### rIPeC attenuated downregulation of HSP70 in the IM after I/R injury

**Figure 8:**
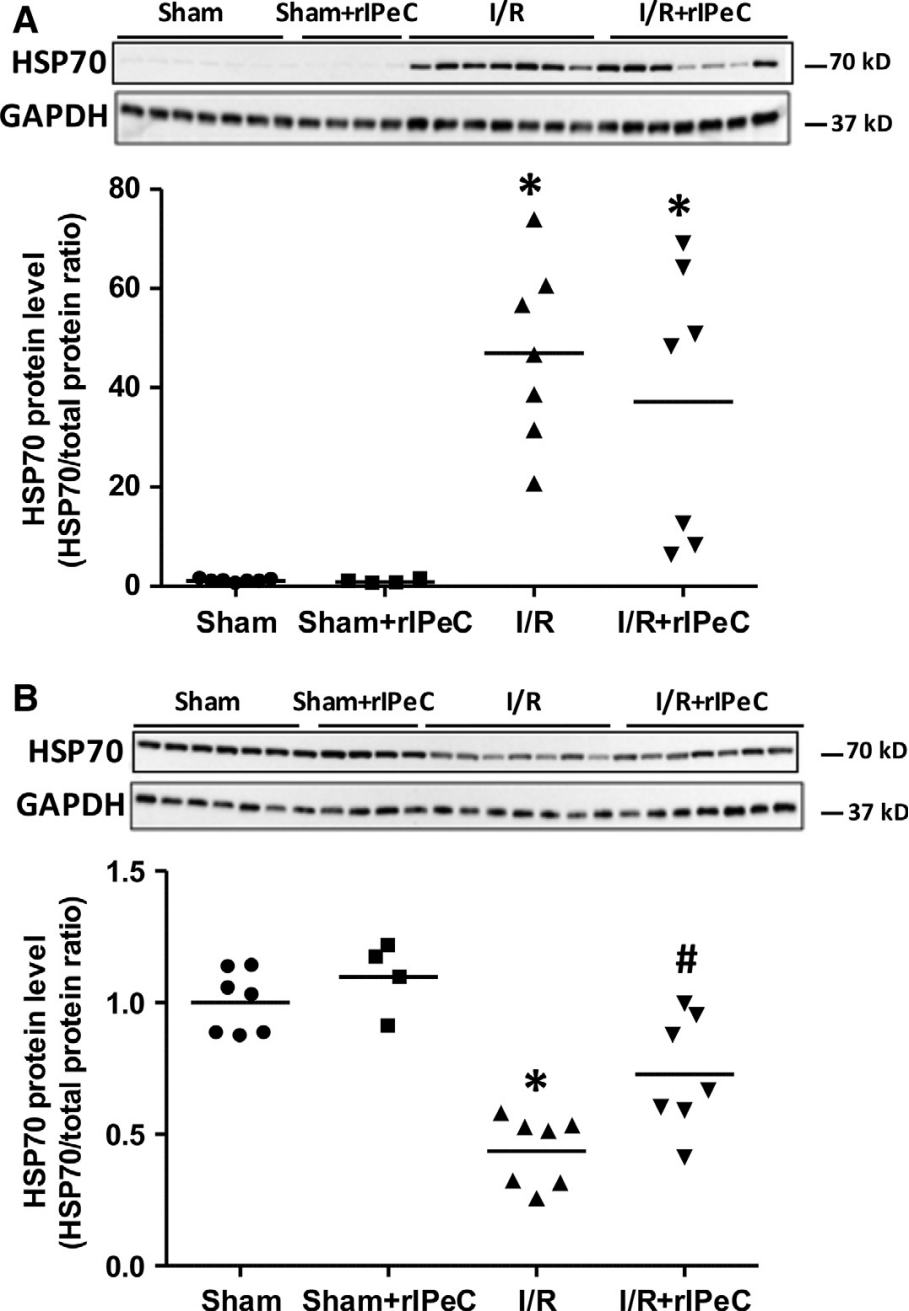
Effects of remote ischemic perconditioning (rIPeC) on heat shock protein 70 (HSP70) expression after ischemia and reperfusion (I/R) injury. Western blot analysis of HSP70 expression in tissue from the cortex and outer medulla (C + OM) (A) and inner medulla (B) in I/R rats treated with rIPeC. Data are means ± SEM, *n *=* *4–8 rats per group for the western blot analysis. *P <* *0.05 compared to the sham group. ^#^ *P *<* *0.05 compared to the I/R group.

In the cortex and OM, HSP70 was significantly upregulated in the I/R group compared to the sham group, and rIPeC treatment did not alter the expression of HSP70 (Fig. 8A). On the contrary, HSP70 was significantly downregulated in IM fractions in the I/R group compared to sham‐operated rats, and this downregulation was significantly attenuated in the I/R + rIPeC group (Fig. 8B).

### Effect of rIPeC on kidney histology

**Figure 9:**
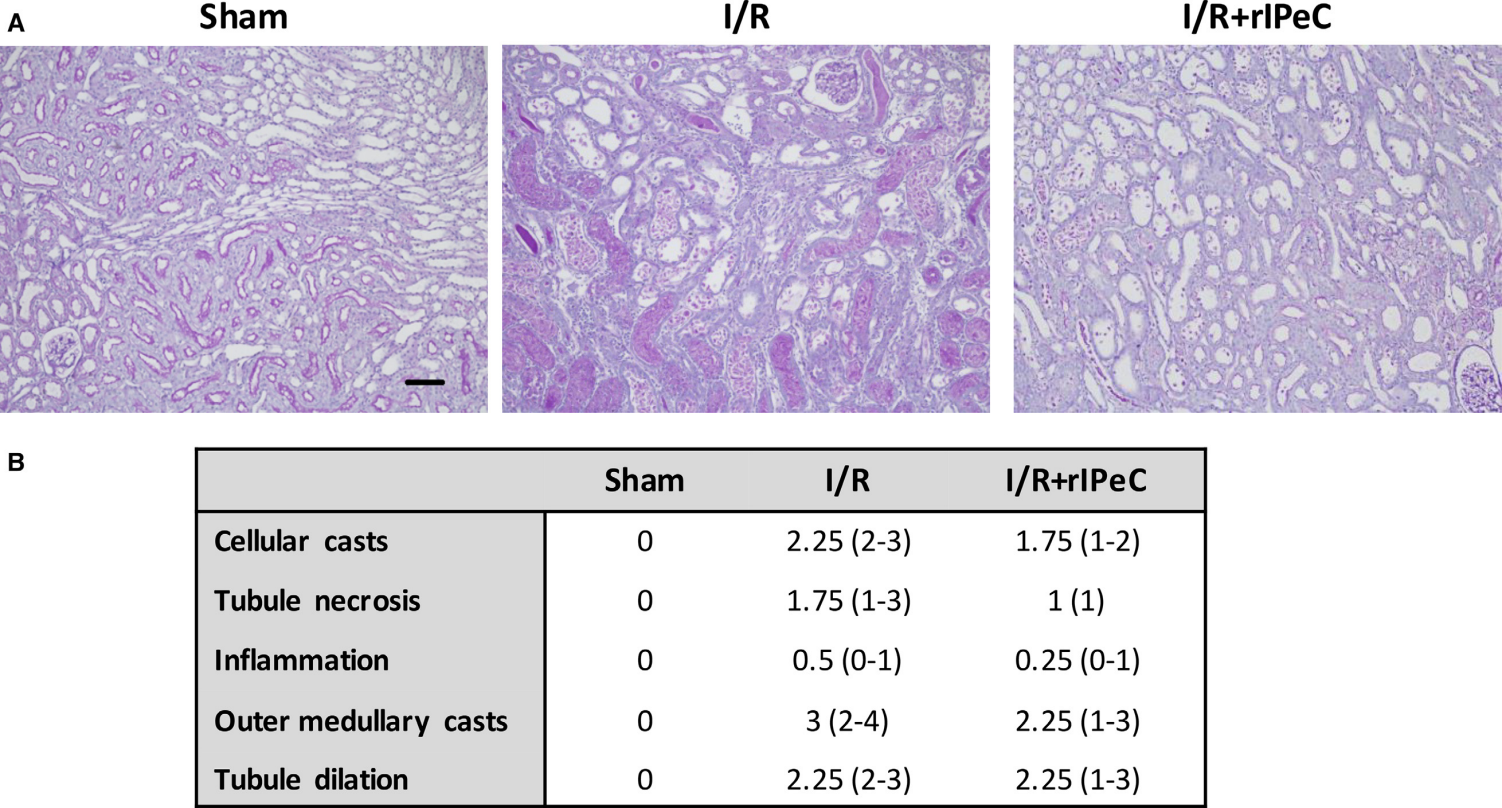
Effects of remote ischemic perconditioning (rIPeC) on renal histological changes after ischemia and reperfusion (I/R) injury. (A) PAS staining of kidneys. Images shown are representative for each group. Scale bar is 100 *μ*m. (B) Histopathological score, evaluated by a skilled pathologist in a blinded manner. Injury scale: 0 = <1%, 1 = 1–5%, 2 = 6–25%, 3 = 26–50%, 4 = 51–100%. *N* = 4 rats per group. PAS, periodic acid–Schiff.

As expected, sections from the sham‐operated rats exhibited no evidence of structural damage (Fig. 9). I/R injury resulted in extensive changes in renal morphology, including destruction of tubules, cast formation, inflammation, and necrosis. The I/R + rIPeC group showed similar ischemic structural changes including cast formation and necrosis, that were, however, less prominent than those seen in I/R rats.

## Discussion

This study demonstrates that remote ischemic *per*conditioning (rIPeC) attenuates the downregulation of AQP2 and phosphorylated Ser256‐AQP2 in the postischemic kidney. This was associated with attenuation of the I/R‐induced decrease in urine osmolality, suggesting that rIPeC reduces ischemia‐induced urinary concentration defects by regulating AQP2 and pAQP2. Previous studies have demonstrated that ischemic preconditioning (IPC) treatment attenuates the decrease in urine osmolality in rats exposed to 45 min of renal ischemia followed by 24 h of reperfusion (Yamashita et al. 2003; Yamasowa 2004). The CD represents the final site of regulated water reabsorption from the tubular fluid and is usually thought to be relatively resistant to ischemic insult (Kortenoeven and Fenton 2014). Nevertheless, it has been demonstrated that I/R injury downregulates CD AQPs (Fernández‐Llama et al. 1999; Gong et al. 2004; Kwon et al. 1999) and that pharmacological intervention using erythropoietin prevents the ischemia‐induced downregulation of AQP2 and improves urinary concentration capability (Gong et al. 2004). This suggests that membrane proteins within CD cells might be affected by I/R injury and by rIPeC. In this study, no effect of rIPeC was observed on the ischemia‐induced downregulation of AQP1, indicating that AQP1 might be less susceptible to the use of rIPeC to treat I/R injury than AQP2.

We found that Na–K‐ATPase expression was significantly reduced in response to renal I/R injury, in agreement with previous studies (Gong et al. 2004; Kwon et al. 1999). In contrast, Na–K‐ATPase activity was not significantly changed between sham‐operated and I/R rats. This was consistent with a study from Molinas et al. (2006) who observed that Na–K‐ATPase activity returned to control level in cortex after ischemia followed by 48 h reperfusion. rIPeC administration tended to increase Na–K‐ATPase activity in I/R kidneys, although we found no statistical difference. This increased activity combined with the tendency to an increased Na–K‐ATPase protein expression level could possibly explain the decrease in FeNa observed in rIPeC‐treated I/R kidneys. Interestingly, our immunohistochemical analysis showed reduced labeling of Na–K‐ATPase in the OM in postischemic kidneys compared to sham kidneys, and this staining appeared considerably stronger after rIPeC treatment. I/R injury also resulted in reduced Na–K‐ATPase fluorescence staining intensity in the injured outer medullary TAL, which was partially reversed in kidneys exposed to rIPeC after I/R injury. Consistent with previous studies, THP staining was mainly found in the cytoplasmic domains of TAL with extension into the lumen in I/R kidneys, while the localization was restricted to the apical surface in the sham kidneys (El‐Achkar et al. 2013, 2008). Interestingly, THP expression was also mostly confined to the apical domains of TAL in the rIPeC‐treated I/R kidneys, suggesting that rIPeC might play a role for the regulation of THP. Indeed, the effect of rIPeC and the molecular mechanisms underlying the targeting of THP requires further investigations.

Na–K‐ATPase creates the driving forces responsible for most ion transport in epithelial cells and is highly expressed in the PT and TAL (Wetzel and Sweadner 2001). It has been suggested that Na–K‐ATPase plays an additional significant role in altered tubular sodium handling after I/R, particularly in the PT (especially in the S3 segment) and the outer medullary TAL, which are known to be the segments most affected by ischemic insult (Alves et al. 2015). In our study, immunohistochemistry showed that Na–K‐ATPase staining seemed weaker in the OM alone in I/R kidneys compared to both sham‐ and rIPeC‐treated I/R kidneys, indicating that regulation of Na–K‐ATPase in response to I/R and rIPeC is more confined to this kidney zone compared to the rest of the kidney. Further analysis of outer medullary Na–K‐ATPase expression revealed that rIPeC showed a tendency to change its expression in the TAL, which to some extent might explain the decrease in FeNa observed in I/R rats subjected to rIPeC treatment.

### Potential mechanisms for the rIPeC‐mediated regulation of Na–K‐ATPase and AQP2 in response to I/R injury

The underlying mechanistic pathways of rIPeC are poorly understood and not yet fully defined. However, it has been postulated that remote ischemic preconditioning (rIPC) involves systemic antiinflammatory, neuronal, and humoral signaling pathways, which may vary in response to different ischemic stimuli and are likely to interact with each other (Kierulf‐Lassen et al. 2015). Heat shock proteins have been identified as playing a pivotal role in rIPC both as a trigger and mediator of the effects of rIPC (Kierulf‐Lassen et al. 2015). Zhang et al. (2008) showed that HSP70 in particular is highly sensitive to I/R injury in rat kidneys. In addition, previous studies have demonstrated that HSP70 is likely to play a role in the localization of Na–K‐ATPase (Aufricht et al. 2002) and in AQP2 trafficking (Park et al. 2013). HSP70 induction is mainly observed in the cortex and OM after I/R (Guo et al. 2014; Schober et al. 1997). In the IM, however, HSP70 levels have been shown to decrease after the initial reperfusion period, indicating zonal changes in HSP70 expression after I/R (Schober et al. 1997). Our data agree with the observations of Schober et al. (1997), who showed marked induction of HSP70 in the cortex and OM, but decreased HSP70 expression in the IM in response to I/R injury.

We found no significant difference in the expression of HSP70 between rIPeC‐treated and ‐untreated I/R rats in cortex and OM tissue. This is consistent with the findings of Guo et al. (2014) who showed that IC had no effect on HSP70 in the cortex and OM after 48 h of reperfusion. However, after 24 h of reperfusion, I/R‐induced HSP70 expression was elevated by ischemic conditioning, indicating that this effect was time dependent (Guo et al. 2014). It has previously been described that IPC is associated with marked cytoskeletal redistribution of HSP70 in the cortex, which can prevent dissociation of Na–K‐ATPase from the cytoskeleton (Aufricht et al. 2002). We did not investigate the cellular but rather the tubular distribution of Na–K‐ATPase and our observations suggest that alterations in the redistribution of Na–K‐ATPase after rIPeC treatment might not be directly dependent on HSP70.

In the IM, HSP70 is expressed mainly in the CD cells (Neuhofer et al. 2005; Park et al. 2013). It has been suggested that HSP70 plays a role in AQP2 trafficking, partly through regulation of AQP2 phosphorylation at Ser256 (Park et al. 2013). Our findings demonstrate that rIPeC prevents downregulation of AQP2 and pAQP2 in rats exposed to I/R injury. This effect was associated with the increased expression of HSP70 in the IM of rats subjected to rIPeC and I/R injury, consistent with the notion that HSP70 may affect AQP2 expression and phosphorylation in response to rIPeC.

As described above, rIPC can trigger a range of other signaling pathways, and considerable interest has focused on the role of protein kinases and transcription factors as important underlying mechanisms of rIPC (Gassanov et al. 2014). Studies have suggested that rIPC can affect several protein kinases, including PKA, PKC, and MAPK (Gassanov et al. 2014) which play important roles for the regulation of AQP2 and Na–K‐ATPase (Gao 2013; Wilson et al. 2013). The underlying mechanisms of rIPeC, however, are poorly understood and it is not known if these kinases are influenced by rIPeC, although it may be speculated that they can contribute to the rIPeC‐influenced regulation of AQP2 and Na–K‐ATPase.

### Renal protective effects of rIPeC

It has been reported that not all combinations and durations of ischemia and reperfusion trigger the conditioning phenomena that protect against I/R injury (Torras et al. 2002). In spite of this, two recent studies have demonstrated that rIPeC using a four‐cycle conditioning schedule of 5 min of ischemia followed by 5 min of reperfusion provides protection against renal I/R injury (Jiang et al. 2015; Sedaghat et al. 2013). Both studies showed that rIPeC improved renal function and structural changes in the kidney after 45 or 60 min of ischemia followed by 24 h reperfusion. We used the same rIPeC schedule, but found no significant improvement of CrCl or BUN in rats exposed to 37 min of ischemia followed by 3 days of reperfusion, indicating that the protection induced by rIPeC can vary according to ischemia and reperfusion time. Despite the nonsignificant improvement in CrCl and BUN, our results indicate that the effects of rIPeC could play a role in the regulation of renal water and sodium excretion by regulating AQP2 expression and phosphorylation and Na–K‐ATPase expression and activity.

In conclusion, the results of this study show a novel effect of rIPeC on the ischemia‐induced control of renal water and sodium excretion, and suggest that regulation of AQP2, pAQP2, and Na–K‐ATPase underlies this effect. Recognizing the potential of rIPeC and understanding its underlying signal transduction may provide an important paradigm for patients presenting with established ischemia.

## Conflict of Interest

The authors declare no conflicts of interest.
